# Anxiety and loneliness among older people living in residential care facilities or receiving home care services in Sweden during the COVID-19 pandemic: a national cross-sectional study

**DOI:** 10.1186/s12877-022-03544-z

**Published:** 2022-12-01

**Authors:** Rose-Marie Johansson-Pajala, Moudud Alam, Annelie Gusdal, Petra von Heideken Wågert, Annica Löwenmark, Anne-Marie Boström, Lena Marmstål Hammar

**Affiliations:** 1 School of Health, Care and Social Welfare, Mälardalen University, P.O 325, SE-63105 Eskilstuna/Västerås, Sweden; 2 School of Information and Engineering/Statistics, Dalarna University, Falun, Sweden; 3 Division of Nursing, Department of Neurobiology, Care Science and Society Karolinska Institute, Stockholm, Sweden; 4 Theme Inflammation and Aging, Karolinska University Hospital, Huddinge, Sweden; 5 R&D unit, Stockholms Sjukhem, Stockholm, Sweden; 6 School of Health and Welfare, Dalarna University, Falun, Sweden

**Keywords:** Aged, Community health services, COVID-19, Emotions, Residential facilities, Social isolation

## Abstract

**Background:**

Older people were subjected to significant restrictions on physical contacts with others during the COVID-19 pandemic. Social distancing impacts older people’s experiences of anxiety and loneliness. Despite a large body of research on the pandemic, there is little research on its effects on older people in residential care facilities (RCF) and in home care services (HCS), who are the frailest of the older population. We aimed to investigate the effect of the first wave of the COVID-19 pandemic in March-May 2020 on experiences of anxiety and loneliness among older people living in RCF or receiving HCS and the impact of the progression of the pandemic on these experiences.

**Methods:**

A retrospective cross-sectional design using data from the national user satisfaction survey (March − May 2020) by the Swedish National Board of Health and Welfare. Survey responses were retrieved from 27,872 older people in RCF (mean age 87 years) and 82,834 older people receiving HCS (mean age 84 years). Proportional-odds (cumulative logit) model was used to estimate the degree of association between dependent and independent variables.

**Results:**

Loneliness and anxiety were more prevalent among the older persons living in RCF (loneliness: 69%, anxiety: 63%) than those receiving HCS (53% and 47%, respectively). Proportional odds models revealed that among the RCF and HCS respondents, the cumulative odds ratio of experiencing higher degree of anxiety increased by 1.06% and 1.04%, respectively, and loneliness by 1.13% and 1.16%, respectively, for 1% increase in the COVID-19 infection rate. Poor self-rated health was the most influential factor for anxiety in both RCF and HCS. Living alone (with HCS) was the most influential factor affecting loneliness. Experiences of disrespect from staff were more strongly associated with anxiety and loneliness in RCF than in HCS.

**Conclusion:**

Older people in RCF or receiving HCS experienced increasing levels of anxiety and loneliness as the first wave of the pandemic progressed. Older people’ mental and social wellbeing should be recognized to a greater extent, such as by providing opportunities for social activities. Better preparedness for future similar events is needed, where restrictions on social interaction are balanced against the public health directives.

## Background

Coronavirus disease 2019 (COVID-19) has spread rapidly worldwide. On March 11, 2020, the World Health organization (WHO) declared COVID-19 a pandemic [1]. The disease poses more of a risk to older than younger people, with more severe disease reported in the older population, including higher mortality and more complications [2]. During the initial months of the pandemic in Sweden in 2020, older people living in residential care facilities (RCF) had the highest excess mortality (75− >100% in April and 25 − 50% in May), followed by those receiving home care services (HCS) (30 − 60% in April and 15 − 40% in May) [3]. Excess mortality in RCF in Stockholm was 11,167 (46%) during March − May 2020 in comparison with the same months in 2016 − 2019 [4]. In contrast, excess mortality was largely unchanged in RCF in several other regions during the same period, with few COVID-19 related deaths reported [5].

Unlike other countries, Sweden did not implement strict regulations, such as quarantine or complete lockdowns, to curb the spread of the virus. Instead, the authorities relied on peoples’ adherence to proposed recommendations, which included limiting social contacts, maintaining good hand hygiene, and avoiding contact with others if experiencing COVID-19 symptoms. The authorities also recommended that people aged 70 years and older should limit their social contacts as much as possible. For example, this age group was advised not to use public transport, shop in stores, or interact with people other than those from their own households. On April 1, 2020, visitor restrictions were implemented nationwide in RCF [6]. In some RCF, physical visits were replaced by social contact via telephone, video calls or through windows. There was concern that social isolation could have negative effects on the psychological and mental health of older people in RCF [2], as previous research has demonstrated detrimental effects of isolation or loneliness on health [7]. Reported health-related effects of isolation or loneliness on older people are physical decline, morbidity, increased mortality, and cognitive and mental health problems, such as depression and dementia, as well as an increased risk of suicide [8 − 10].

A growing body of evidence points to an impact of COVID-19 on psychological and mental health in the older population during the first months of the pandemic (March − June 2020). Several studies report an increased incidence of anxiety, depression, and loneliness in this population [11 − 13]. Studies also suggest that cognitive functions deteriorated in persons with dementia, with a rise in behavioural and psychological symptoms of dementia, including symptoms of anxiety and depression [14, 15]. Other studies have presented contrasting findings, suggesting that older people seem to be more resilient to anxiety and depression than younger people, at least during the first wave of the COVID-19 pandemic [16 − 18]. Kivi et al. [19] even reported lower levels of worry and higher well-being among older people in Sweden than in previous years. From a European Union (EU) perspective, the psychological impact of COVID-19 was particularly severe among those aged 80 and older, with 23% reporting feeling sad or depressed more often and 18% reporting feeling lonelier than before the pandemic by the summer of 2020 [20].

Despite a large body of research on the pandemic, there is little research on its effects on subgroups of older people, including those living in RCF and those receiving HCS [2]. This poses a risk of excluding older people with limited resources. Such persons are least likely to use technical devices, such as social technology, to stay connected to other people and are the most vulnerable to loneliness [21]. A research report based on EU survey data on the impact of the COVID-19 pandemic on the quality of life of older citizens concluded that further research on older people in vulnerable situations is needed. The report state that older people in residential care are often excluded from surveys and that further research on this population is warranted [20].

Besides being particularly vulnerable, older people living in RCF or receiving HCS are dependent on staff. This poses an additional risk, as previous research shows that RCF and HCS staff had insufficient personal protective equipment, low testing capacity, and were working while experiencing mild COVID-19 symptoms [13, 22]. Maintaining hand hygiene with the aim of preventing the spread of the virus is problematic in RFS and HCS due to a lack of appropriate training of staff. Their main responsibilities are domestic tasks, assistance with household tasks and personal care, such as bathing and dressing [23]. Furthermore, the staff often lack an appropriate education and are employed on a per-hour basis, resulting in high staff turn-over and thus a higher risk of virus spread [24]. Registered nurses have the highest medical competence, but they only constitute about 10% of the staff [25].

The impact of COVID-19 is expected to vary across countries and age-specific subpopulations [26]. Thus, it is important to study the impact of COVID-19 at national levels and in subgroups of older people, such as those living in RCF or receiving HCS. In Sweden, an annual survey conducted by the National Board of Health and Welfare (NBHW) revealed a decrease in anxiety and worry among older people receiving HCS in 2020 as compared to 2019, whereas older people living in RCF experienced more anxiety and worry and were lonelier in 2020 than in 2019 [27]. However, this report did not focus on specific risk factors for loneliness and anxiety in RCF and HCS settings or differences in experiences as the pandemic progressed.

It is important to investigate older peoples’ experiences of daily life in RCF and HCS settings during the pandemic to be better prepared for possible similar pandemics in the future. Such research is also needed to obtain unique information on these vulnerable groups’ perceptions in regard of differences in experiences related to living in RCF or receiving HCS. The aims of this study were to investigate the effect of the first wave of the COVID-19 pandemic (March-May 2020) in Sweden on experiences of anxiety and loneliness among older people living in RCF or receiving HCS and the impact of the progression of the pandemic on these experiences.

## Methods

### Study design

This study used a retrospective cross-sectional design. Data were retrieved from a national survey by the NBHW [28] and from two national databases, the National Patient Register and National Prescribed Drug Register, also maintained by the NBHW. The NBHW received the completed survey forms between March 9 and May 25, 2020, although some survey forms were not returned until the first week of June. The data collection period coincided with the rising phase of the first wave of COVID-19 in Sweden. This study exploited the spatio-temporal variation in the progression of COVID-19 spread during the data collection period to identify the effect of COVID-19 on the experience of anxiety and loneliness. It was assumed that the exact response time (week) of the survey was completely at random. The NBHW survey data were collected from older people in RCF and HCS settings, and these two groups were treated as two independent populations.

### Setting and sample

The RCF and HCS study populations consisted of 70,077 and 145,445 persons, respectively, who received care services in 2020. All those living in RCF or receiving HCS in 2020 were invited to participate in the survey. The response rate was 57% and 40%, respectively. The non-response mechanism was assumed to be missing completely at random (MCAR) [29], and the plausibility of the assumption was verified using descriptive statistics. Relying on the MCAR [29] assumption, the responses were considered a random sample of the respective population.

### Data collection

This study utilized data collected by the NBHW [27] as part of its annual quality assessment of RCF and HCS. The NBHW survey consists of structured questionnaires disseminated via the regular postal service. The respondents could either return filled in survey forms via regular post, or they could answer the survey over internet using a weblink provided in the survey form. The NBHW survey questionnaires for RCF and HCS consist of 28 and 23 questions, respectively. The survey questions covered various common concepts, for example, health and mobility status (self-assessed); influence; provision of support and help; treatment by staff; security; anxiety; availability and overall perception of RCF/HCS. The RCF questionnaire contains an additional section on mealtimes and the interior and exterior environments. The responses to questions in this section were not included in the present study. One question on loneliness and one question on anxiety were used as the response (dependent) variables. Health status, mobility, treatment by staff, living with an adult (dummy) and a proxy response (dummy) were used as independent variables. A substantial proportion of the responses (83% and 41% for RCF and HCS, respectively) were proxy responses (i.e., a relative or member of staff completed the survey questionnaire, often together with the intended respondent). Both the self-responses and proxy responses were included in this study.

To identify persons with dementia, we used medical register data on diagnosis and medication, also maintained by the NBHW. Persons with a diagnosis of dementia in 2020 were identified using ICD-10 codes F00-F03 or had been prescribed medication using code N06D. Persons without these codes were considered not to have dementia. Data on age, sex and region of residence were obtained from the patient register data base, also maintained by the NBHW. Information on weekly COVID-19 infection rates was obtained from the official website of the Swedish Public Health Agency [6]. A description of the dependent and independent variables is given in Table 1.

**Table 1:** Dependent and independent variables used in the study

Variable	Question/Description	Source	Response options/Scale of measurement
*Dependent variables*			
Anxiety	Are you troubled by anxiety, worry or anguish?	Survey	1 = No, 2 = Yes, slightly, 3 = Yes, severely
Loneliness	Does it happen that you are troubled by loneliness?	Survey	1 = Yes, often, 2 = Yes, now and then, 3 = No
*Independent variables*			
Age	Age of the NBHW survey respondent	Register	Age in years
Sex	Sex of the respondent	Register	1 = Male, 2 = Female
Dementia	Whether the respondent was diagnosed with dementia in 2020	Register	ICD-10 codes F00-F03; medication code N06D
Self-rated health	How would you describe your general health status?	Survey	1 = Very good, 2 = Quite good, 3 = Fair, 4 = Quite poor, 5 = Very poor
Household status	Do you live with another adult? (only for HCS)	Survey	1 = Yes, 2 = No
Disrespect	Did you experience any negative incidences relating to a staff member (nine listed items) in the last year?	Survey	1 = Yes, 5 = No
Mobility	How is your in-house mobility?	Survey	1 = I can move alone, 2 = I have some difficulty in moving, 3 = I have great difficulty in moving alone, 4 = I cannot move alone
Proxy	Who helped you complete the questionnaire?	Survey	1 = Older person, 2 = Proxy (relative, friend, personnel, etc.)
Infection	COVID-19 infection rate in the preceding 2 weeks in the respective county	Internet	Confirmed infection rate per 100,000 persons in the preceding 2 weeks in the respective county
Region	Region that ordered the HCS/RCF service	Register	21 counties (regions) of Sweden

*Note*: Register refers to the patient register, and medical register maintained by the NBWH, survey refers to the Annual Quality Evaluation Survey 2020 maintained by the NBHW, and Internet refers to the official website of the Swedish Public Health Agency.

### Data analysis

As the dependent variables, anxiety and loneliness, are ordinal variables (Table 1), we used a proportional odds (PO) cumulative logit model [30] for estimating the strength of association of the independent variables with the dependent variables and for drawing inference on those association measures. These models were fitted separately for the RCF and HCS respondents. As these two response groups are different with respect to average age, health status and prevalence of dementia and other comorbidities, they were treated as two separate populations. In addition, there were marked differences between the living conditions of the two groups, with the RFC group subjected to greater restrictions than the HCS group in terms of interactions with family and friends. Each response variable was fitted in an independent PO model with the same set of independent variables, except household status (i.e., living with another adult), which did not apply to RCF respondents and thus was not included in the survey questionnaire. The PO model was fitted using the ‘polr’ function in the MASS library [30] in R statistical software [31]. The degree of association between the independent and dependent variables was estimated in terms of cumulative odds ratio (COR), with statistical inferences drawn at the 95% confidence interval level of the COR parameter and a significance test of the respective log (COR) parameter at the 5% level of significance. All the data manipulation and analysis were performed using R [31].

### Ethical considerations

Informed consent was obtained from all respondents by the NBHW. Data received from the NBHW were coded and no respondent could be identified in the study. Respondents were informed by the NBHW that their data could be used in research. The study was approved by the Regional Research Ethics Committee of Uppsala, Sweden (Reg. No. 2017/140). Ethical standards for scientific work were followed and based on guidelines and regulations [32].

## Results

### Sample characteristics

From the descriptive statistics of the dependent and independent variables (Table 2), compared to the HCS respondents, the RCF respondents were older, had poorer self-rated health, worse mobility conditions, higher dementia prevalence and lower mobility status. In both RCF and HCS response groups females, and the older persons (aged 80 years and above) were overrepresented in the sample. The RCF respondents experienced more anxiety and loneliness than the HCS respondents. In both groups, loneliness was more prevalent than anxiety. About 50% of the respondents were from the three largest (in terms of population) regions in Sweden, which together is home to 53% of the total Swedish population. This indicates that the number of respondents in each region was proportional to the population size (approximately) of the region, which is expected when the non-response mechanism is MCAR.

**Table 2:** Descriptive statistics of the study variables

Variable	Respondent groups	
	Residential care facility (RCF) *(n* = 27,872)	Home care service (HCS) *(n* = 82,834)
*Dependent variables*	Percentage (counts in parenthesis)	Percentage (counts in parenthesis)
Anxiety		
3 = Yes, severely 2 = Yes, slight 1 = No	12% (3,316) 51% (13,869) 37% (9,952)	6% (5,096) 41% (32,972) 53% (43, 461)
Loneliness		
1 = Yes, often 2 = Yes, sometimes 3 = No	19% (4,696) 50% (12,171) 30% (7,356)	13% (9,738) 40% (31,019) 47% (36,901)
*Independent variables*		
Proportion of respondents aged older than 80 years	80% (count = 22,208, mean age: 87 years)	71% (count = 58,677, mean age: 84 years)
Proportion of females	68% (18,871)	65% (53,932)
Prevalence of dementia	21% (5,739)	8% (6,232)
Self-rated health: Poor or worse	31% (8,622)	22% (17,869)
Household status: Living alone	-	74% (59,717)
Disrespect (exposure %)	24% (6,172)	16% (12,700)
Mobility: Unable to move unaided or great difficulty in moving	82% (22,677)	72% (58,692)
Proxy response	64% (17,565)	31% (23,995)
Respondents from three major regions, Stockholm, Skåne and Västra Götaland (% of total in 21 regions)	47% (13,007)	49% (40,595)

Note: The difference between the sample size (n) and the sum of the counts of the response alternatives gives the frequency of item non-response. For example, for “Anxiety” among RCF respondents, 27,872-(3316 + 13,869 + 9952) = 735 shows 735 missing observations this variable. The percentages, and the means are calculated based on the non-missing observations in the respective variable, e.g. for Anxiety (3 = Yes, severely) in RCF, 3316/(3316 + 13,869 + 9952) = 0.1222 which is rounded to 12%

### Anxiety and loneliness during the COVID-19 pandemic

Regarding the effect of the progression of the pandemic, the PO models (Table 3) revealed that as the infection rate (per 100,000 population) increased by 1%, the cumulative odds of feeling anxiety increased by 1.06% and 1.04% among the HCS and RCF respondents, respectively, assuming that all other independent variables remained constant. Similarly, the cumulative odds of feeling (often to sometimes) lonely increased by 1.13% and 1.15% among the HCS and RCF respondents, respectively, in accordance with a percentage change in the infection rate. The following variables were associated with an increased level of anxiety and loneliness: female sex, dementia, poor health, living alone and experiences of disrespect. For both RCF and HCS respondent groups, older respondents (aged 80 years and above) felt less anxiety but more loneliness, although the age effect on loneliness was insignificant for HCS respondents. Self-rated health was the most influential factor for anxiety, with both RCF and HCS respondents with poor health having 3.9 times higher cumulative odds of feeling greater (severe to slight and slight to no) anxiety. Household status (living alone) was the most influential factor affecting loneliness, increasing the COR by a factor of 3.9. In contrast, living alone increased the COR for anxiety only by 5%. Thus, compared with the HCS respondents living with another adult, those living alone had 3.7 times higher (approximately) cumulative odds of a higher degree of loneliness than higher anxiety. Although the cumulative odds for loneliness were also high, compared with the respondents with good health, those with poor health had 1.7 times (approximately) higher cumulative odds of a greater degree of anxiety than loneliness, both in the RCF and HCS groups.

The fitted PO model (Table 3) revealed that the direction and strength of the association between the independent and dependent variables (anxiety and loneliness) were similar between the HCS and RCF respondents, except for the effect of age on loneliness, mobility and region on anxiety, and disrespect on both dependent variables. Other than these four exceptions, the 95% confidence intervals of the CORs overlapped between the RCF and HCS respondents, indicating that these two groups’ opinions did not differ significantly on most of the issues included in this study. In the PO model using anxiety as the dependent variable, the effects of mobility and region were significant for HCS respondents but insignificant for RCF respondents. Furthermore, within each facility (RCF and HCS), the effects of the independent variables on anxiety differed by a higher degree from that on loneliness (Table 3; compare columns 1 vs. 3, and 2 vs. 4) than they did between the two types of facilities (compare columns 1 vs. 2, and 3 vs. 4) for a given dependent variable. The effect of the proxy responses was negative and significant, indicating that the proxies (relatives or family members) considered, on average, that the respondents felt lonelier and more anxious than the self-respondents reported.

**Table 3:** Cumulative odds ratios (CORs) (95% confidence intervals in parenthesis) from the fitted proportional odds (PO) models for loneliness and anxiety among Home Care Service (HCS) and Residential Care Facility (RCF) respondents

Effects	Anxiety (Response order: Severe < Slight < None)		Loneliness (Response order: Often < Sometimes < Never)	
	**HCS**	**RCF**	**HCS**	**RCF**
Age (ref: < 80 years)				
Older than 80 years	0.81 (0.78, 0.84)	0.88 (0.83, 0.94)	1.03^ns^ (0.99,1.06)	1.14 (1.07, 1.22)
Sex (ref.: male)				
Female	1.44 (1.39, 1.49)	1.38 (1.30, 1.45)	1.19 (1.15, 1.23)	1.11 (1.05, 1.17)
Dementia (ref.: no dementia)				
Persons with dementia	1.26 (1.19, 1.33)	1.35 (1.26, 1.43)	1.43 (1.35,1.51)	1.18 (1.10, 1.26)
Self-rated health (ref.: not poor)				
Poor or very poor	3.89 (3.74, 4.04)	3.93 (3.70, 4.10)	2.23 (2.15, 2.31)	2.22 (2.09, 2.35)
Household status (ref.: living with an adult)				
Living alone	1.05 (1.01, 1.08)		3.86 (3.71, 4.01)	
Disrespect (ref.: experienced)				
No experience of disrespect	1.60 (1.54, 1.67)	2.17 (2.04, 2.30)	1.62 (1.55, 1.68)	3.17 (2.98, 3.38)
Mobility (ref.: Can move alone)				
Unable to move unaided or great difficulty in moving	1.32 (1.27, 1.36)	1.02^ns^ (0.96, 1.09)	1.33 (1.28, 1.37)	1.27 (1.18,1.36)
Survey responder (ref.: self)				
With the help of (or by) a proxy	1.48 (1.43, 1.53)	1.49 (1.42, 1.57)	1.67 (1.62, 1.73)	1.55 (1.46, 1.63)
log (COVID-19 infection in the last 2 weeks/100 th. population)	1.06 (1.04, 1.08)	1.04 (1.01, 1.07)	1.13 (1.11, 1.15)	1.15 (1.12, 1.18)
Region (21 counties in Sweden; dummy variable)^1^	Yes	Yes^ns^	Yes	Yes

*Notes*: ^1^20 dummy variables for 21 Swedish regions were included (one less due to identifiability), but all the 20 region effects are not reported to keep the table short. ^ns^Not significant at 5% significance level. A COR of > 1 indicated higher odds of answering towards a lower response category for an increase in the effect variable, and vice versa

## Discussion

Many studies worldwide have reported the impact of the first wave of the pandemic on the psychological and mental health of the older population. The contributions of our study are as follows: It is a national study, including more than 110,000 of the oldest of older people (majority > 80 years old) in Sweden. It also focuses on the experiences of two specific groups (i.e., those living in RCF and those receiving HCS) of anxiety and loneliness from the initial stages of the pandemic through progression and spread, between early March and end of May. No previous studies seem to have addressed the impact of the progression of the pandemic on experiences of loneliness and anxiety in this population.

The results showed that older people in both the RCF and HCS groups experienced more loneliness than anxiety and that these experiences increased in accordance with the progression of the pandemic. Both anxiety and loneliness were more prevalent among the RCF than HCS respondents. Other studies conducted on older populations (not RCF or HCS) in other countries during the same period (March − May 2020) also reported a rise in the prevalence of loneliness. For example, studies in the United States, the Netherlands, Spain and the United Kingdom found increased or high levels of loneliness during this period [11, 13, 16, 18, 33]. In contrast, studies in other countries, for example, Germany and Austria, suggest that experiences of loneliness did not increase during March − May 2020 [34, 35]. Similar variation was found in terms of anxiety, with a study in the United Kingdom reporting only a slight increase in anxiety compared to the pre-pandemic period [12]. Studies in Spain, Canada, Austria and Israel also suggested that older people did not seem to be especially vulnerable in terms of the development of anxiety during the initial phase of the pandemic [16, 17, 36 − 40]. However, it is difficult to draw comparisons between the findings of these studies due to the different ways in which the respective countries responded to the pandemic. Most countries implemented considerably stricter regulations than Sweden did. Moreover, methodological differences, biases in survey designs and differences in the study populations, for example the age of the population, mean the findings are not directly comparable. However, the factors found to influence anxiety and loneliness in the present study were consistent with those found in previous research conducted during the same period. Females reported more anxiety and loneliness than males, which is in accordance with previous reports [37, 41 − 43]. Living alone had a significant impact on feelings of loneliness, as found in several studies [4, 43, 44]. In addition, in accordance with the literature [14, 15], persons with dementia experienced more anxiety.

Most of the independent variables included in the present study were negatively associated with experiences of anxiety and loneliness. The variable that contributed the most to anxiety in both the RCF and in HCS groups was poor self-rated health, and its effect on loneliness was also high. Of note, experiences of disrespect by staff were associated with increased anxiety and loneliness among both the RCF and HCS respondents but to a significantly higher extent in the RCF. A decrease in satisfaction due to not being treated with dignity and respect in HCS was reported already before the pandemic [45]. As in the present study, poor-rated health and dementia were important factors. A cross-sectional study focusing on RCF found similar results [46]. Considering the aforementioned findings in the pre-pandemic period, the findings of the present study on the impact of disrespect by staff on feelings of loneliness and anxiety during the pandemic are not surprising.

A report by the NBHW revealed a decrease in anxiety and worry among older people receiving HCS between 2019 and the first wave of the pandemic in 2020, whereas those living in RCF experienced more anxiety and worry and were lonelier in 2020 than in 2019 [27]. We did not compare the data in 2020 with data for 2019. Instead, we analysed differences in the responses of the RCF and HCS groups to questions about anxiety and loneliness as the pandemic progressed. According to our results, although experiences of anxiety and loneliness were more common in RCF than HCS settings, loneliness and anxiety increased significantly in both settings as the pandemic progressed.

There are several possible explanations for the increased prevalence of anxiety and loneliness in RCF as the pandemic progressed. First, those living in RCF are generally frailer and have more complex health problems than those receiving HCS. Therefore, they have an elevated risk of developing severe symptoms of COVID-19, with a subsequent increase in mortality. In our study, poor self-rated health was a strong influential factor for anxiety and loneliness. According to the literature, about half of all COVID-related deaths during the first wave of the pandemic occurred in RCF [22, 47, 48], with the highest excess mortality in Sweden occurring in RCF during this period [3]. Thus, it might not be surprising that residents in RCF are likely to experience more anxiety and loneliness than those living in their own homes. However, a previous study suggests that being isolated from the outside world could bring a sense of security to older people in RCF in terms of virus transmission and receiving support by staff in daily living [49]. Based on the findings and previous studies, it can be assumed that experiences varied significantly due both to the progression of COVID-19 in various regions and in specific RCF. Older people living in their own homes have described experiences of fear when leaving their homes yet feeling like prisoners in their own homes [50]. However, home-dwelling elders in Sweden were not subjected to visitor restrictions, only recommended to limit their contacts with other people as much as possible, recommendations with which they complied [51].

Second, visitor restrictions implemented in RCF likely influenced experiences of anxiety and loneliness [6]. Moreover, some residents were unable to go outside or even leave their rooms during the pandemic [52]. Although digital technology, such as video calls, partially replaced physical visits and social contacts [43], this was probably not possible for all residents. As loneliness and lack of physical contact are risk factors for both physical and mental illnesses, it is important to plan for future similar events [35]. Digital technologies have the potential to alleviate loneliness and social isolation in older people [53–55]. Therefore, RCF should be equipped with a basic technology infrastructure to facilitate modern-day technologies, which can provide opportunities for social connections [56]. In addition to possibilities for connecting family and friends, opportunities should be created to enable various activities, such as physical activities, which have been shown to be directly related to decreased anxiety symptoms in older people [57]. As physical and cognitive impairment (e.g. dementia) are common among the oldest members of society, particularly those in RCF, they may not have the ability to use specific technologies independently. Therefore, the technologies need to be well designed and easy to use, and the staff needs skills in how to support older people. Of course, it is important to note that even if technology can enable social connectedness and activities, it cannot replace physical contact.

Third, issues pertaining to staff could have contributed to experiences of anxiety and loneliness among those in RCF. To prevent the virus spreading, the staff were required to wear protective equipment (e.g., visors, googles and mouth guards) and to minimize social interactions. According to a previous study, such measures create a distance and severely affect the mental well-being of residents [58]. The ability of staff to adhere to routine hygiene protocols was also an issue. According to a report on RCF in Sweden, the availability of protective equipment and adherence to hygiene routines were poorer in RCF with a spread of COVID-19 [59]. Most staff are not sufficiently trained in hygiene routines, and they often lack an appropriate education [23, 24]. Circumstances such as those mentioned above have been widely reported by the media, which might have increased the level of anxiety among older people. Headlines of failures and fatalities have been described to cause distress among both older people living in RCF and their families [60]. Older people receiving HCS could refuse help from staff, which some did during this time [61], and manage with the help of relatives. In contrast, those living in RCF were dependent on staff to provide support with daily care.

Measures are needed not only to ensure better preparedness in the future for new pandemics or other severe events but also because improvements are required in general in the care of older people. As suggested by Chu et al. [56], administrators and health care professionals must move beyond reactionary responses and towards proactive and thoughtful consideration of how care for older people (RCF and HCS) can be best supported in the future. Staff must be adequately trained and have access to proper equipment to provide safe, good-quality care. It is imperative that RCF and HCS staff recognize the older people’s psychological and emotional needs. Previous research has demonstrated detrimental effects of isolation or loneliness on health [7–10]. Therefore, restrictions concerning, for example, opportunities to see family members, should be balanced with the public health imperative. Moreover, registered nurses specialized in geriatric care need to be more involved in everyday care in RCF and HCS to support staff in providing good-quality care to older people.

### Strengths and limitations

One strength of this study is the access to a large nationwide survey, which was conducted around the time of the first wave of the COVID-19 pandemic in Sweden, and the access to relevant national register data. Therefore, the sample may be regarded as representative, which strengthens the generalizability of the study. One limitation is the use of a single item to measure loneliness and anxiety. However, the study is based on an annual survey conducted by the Swedish National Board of Health and Welfare, which do not include any additional measurements. Other limitations are the survey non-response rate, which was higher in 2020 by 3% and 10% [27] for RCF and HCS, respectively, and the relatively lower rate of proxy response compared to 2019. As a result, those with relatively poor self-rated health and older age might have been underrepresented in the 2020 NBHW survey. As no information about the non-response was available, it was not possible to conduct a thorough investigation into the missing data mechanism. Moreover, the dementia status was based on diagnosis and medication records, which can be a reason for that the prevalence of dementia is probably higher than reported in the study. Nor are everyone with dementia diagnosed or receive medication treatment. Another possible limitation is that the data were collected in a short period in the initial stage of the pandemic and covered just one infection wave. However, Sweden had the highest excess mortality in RCF and HCS settings during this initial stage (March-May 2020) [3].

## Conclusion

Older people in RCF or receiving HCS experienced increasing levels of anxiety and loneliness as the first wave of the COVID-19 pandemic progressed. The mental and social well-being of older adults should be recognized to a greater extent, such as by providing opportunities for safe social connectedness and activities. Strategies are needed to ensure better preparedness for future pandemics or other similar events, where restrictions on social interaction are balanced against the public health directives.

## Data Availability

The data presented in this study are available on request from the corresponding author. The data are not publicly available due to restrictions in accordance with the Swedish Public Access to Information and Secrecy Act (2009:400). Requests for access to the dataset should be sent to the corresponding author and will be considered by the university’s data protection officer.

## Abbreviations

COVID-19: Coronavirus disease 2019
HCS: Home care service
RCF: Residential care facility
